# Resistive Oxygen Gas Sensors for Harsh Environments

**DOI:** 10.3390/s110403439

**Published:** 2011-03-24

**Authors:** Ralf Moos, Noriya Izu, Frank Rettig, Sebastian Reiß, Woosuck Shin, Ichiro Matsubara

**Affiliations:** 1 Bayreuth Engine Research Center, University of Bayreuth, Bayreuth 95440, Germany; E-Mails: frankrettig74@googlemail.com (F.R.); sebastian.reiss@uni-bayreuth.de (S.R.); 2 Advanced Manufacturing Research Institute, National Institute of Advanced Industrial Science and Technology (AIST), Nagoya 463-8560, Japan; E-Mails: n-izu@aist.go.jp (N.I.); w.shin@aist.go.jp (W.S.); matsubara-i@aist.go.jp (I.M.)

**Keywords:** strontium titanate, ceria, titania, lambda-probe, UEGO, conductometric gas sensors

## Abstract

Resistive oxygen sensors are an inexpensive alternative to the classical potentiometric zirconia oxygen sensor, especially for use in harsh environments and at temperatures of several hundred °C or even higher. This device-oriented paper gives a historical overview on the development of these sensor materials. It focuses especially on approaches to obtain a temperature independent behavior. It is shown that although in the past 40 years there have always been several research groups working concurrently with resistive oxygen sensors, novel ideas continue to emerge today with respect to improvements of the sensor response time, the temperature dependence, the long-term stability or the manufacture of the devices themselves using novel techniques for the sensitive films. Materials that are the focus of this review are metal oxides; especially titania, titanates, and ceria-based formulations.

## 1. Introduction

**Figure 1.**
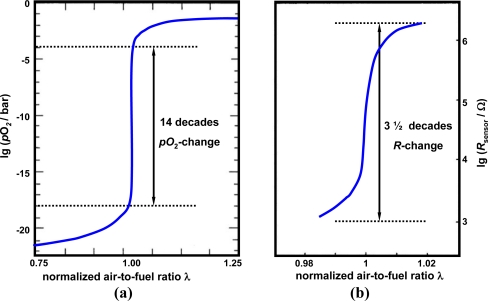
**(a)** Relationship between oxygen partial pressure and normalized air-to-fuel ratio *λ* (modified after [6]). **(b)** Behavior of the sensor resistance *vs*. normalized air-to-fuel ratio *λ* (modified after [10]) around the stoichiometric point at *λ* = 1.

The story of the resistive oxygen gas sensor started in the 1960s when exhaust gas after-treatment concepts for reducing pollutants from automotive exhausts were suggested. In order to ensure an optimized catalyst efficiency with respect to hydrocarbons, carbon monoxide, and nitrogen oxides, the engine has to be operated stoichiometrically at *λ* = 1, with *λ* being the normalized air-to-fuel-ratio. Only at *λ* ≈ 1, can the so-called three-way catalyst convert all limited emissions. For further information on automotive exhaust catalysts, see the overview in Reference [1]. Since the oxygen partial pressure varies by approx. 14 to 16 decades around the stoichiometric point [Figure 1(a)], amongst others, measurement of the oxygen partial pressure, *p*O_2_, of the exhaust gas for engine control purposes was suggested. Two sensor concepts emerged. The first concept, which prevails nowadays, uses zirconia concentration cells (*λ*-probes). They measure the oxygen concentration *versus* an air duct, which serves as an oxygen reference. Since the concentration cell provides a voltage of about 50 mV per decade *p*O_2_-difference, a strong voltage change occurs around *λ* ≈ 1. For applications in a broad air-to-fuel range, zirconia pumping cell sensors were developed by all major sensor manufacturers. Even a combined NO_x_/O_2_ sensor is on the market. Reference [2], which is written from an industrial point of view, gives an overview on this technology. It also considers the interactions between sensor development, automotive requirements, and legislative regulations. The current development status of the zirconia *λ*-probe is described in Reference [3].

The second concept uses the *p*O_2_-dependent resistance of metal oxides for *λ* determination, which is the focus of the present review. Since the resistivity of almost all semiconducting metal oxides depends on the oxygen partial pressure of the surrounding gas atmosphere, most metal oxides are in principle suitable as materials for high temperature resistive oxygen sensing.

An entire overview would go far beyond the scope of a review article. Therefore, this device-oriented review article concentrates on some key materials, which are oxides based on TiO_2_, SrTiO_3_, Ga_2_O_3_, and CeO_2_, with a special focus on exhaust gas oxygen sensing applications in harsh environments. In the past, several groups worked on concepts to remove the temperature dependency of the resistivity of the utilized semiconducting sensor materials. These approaches will be highlighted as well in this review.

## 2. Titania Sensors

Some of the first investigated resistive gas sensors were based on titania (TiO_2_). For instance, the resistance of titania sensors increases monotonically in the *p*O_2_-range between 10^−24^ bar and approximately 10^−2^ bar [4]. It follows Equation 1, wherein *A*_0_ denotes a constant and *E*_A_ is the thermal activation energy of the electrical conductivity:

(1) $$R_{\mathrm{sensor}}=A_{0}\cdot \exp\left(\frac{E_{A}}{kT}\right)\cdot {\left(p0_{2}\right)}^{m}$$

This *R*(*p*O_2_*^m^*)-dependence can be explained by bulk defect chemistry, eventually leading to Equation 1 (e.g., see the textbook [5]). With increasing oxygen concentration in the ambient gas, *i.e.*, when increasing *p*O_2_, oxygen vacancies fill up (or the concentration of titania interstitials diminishes, which leads to the same resistivity behavior from the defect chemistry point of view). The result is a smaller concentration of mobile electronic charge carriers and therefore an increase of the electrical resistivity with increasing *p*O_2_. The exponent *m* for titania is in the range between 0.2 and 0.25.

Due to the steep *p*O_2_-change around *λ* ≈ 1 [Figure 1(a)], the sensor resistance varies by several decades around the stoichiometric point [Figure 1(b)]. The first attempts to realize such sensors were conducted by the Ford Motor Company (e.g., see [6]). Ceramic titania pellets were used. A small addition of donors extended the monotonically region to 0.2 bar (air). A few years later, titania thick-film sensors were investigated by several companies (e.g., [7,8]) and constantly improved [9]. NGK-NTK has developed a serial product based on this concept. These sensors are produced in a hybrid technology using screen-printed thick-films on ceramic tapes that are co-fired. A comprehensive summary on this sensor is given by Takami [10]. Up to now, this has been the only successful attempt to bring titania thick-film resistive exhaust gas oxygen sensors in serial production for automotive applications. A comprehensive overview showing the status of the development as of 1988 is given in [11].

While in the 1990s research interests moved from titania to other oxide sensor materials (see below), titania has again become a research topic of interest and today several groups are working on resistive titania oxygen sensors. Their research is directed in several directions.

First of all, the application at lower sensor temperatures is under study. At lower temperatures, the sensor response is typically not volume-controlled anymore, but becomes surface-controlled. In other words, the sensors respond markedly to oxidizing and reducing gases, like NO_2_ or CO, respectively. Details can be found, e.g., in the review [12]. In order to reduce these cross responses and to enhance the oxygen surface reaction, a catalytic activation is helpful. For that, the sensors are activated by applying finely dispersed platinum salt solutions into the porous films. This is an already known and widely used procedure (see, e.g., References [11,13,14]), but recently, it has been shown that the high temperature treatment after the platinum addition is the crucial parameter for the sensor kinetics [15], since it influences the oxygen exchange rate between oxide material and gas phase. Sensor response times in the range of a few tens of milliseconds can be reached, which is in the same order of modern zirconia concentration cells. By applying porous catalytic filters on top the sensor, its behavior can be strongly modified, especially its response to CO becomes prominent [13,16].

In a second research direction, sensors manufactured with novel preparation techniques are under study. During the high-temperature firing processes, there is always an interaction of the sensor material with the alumina substrate. This may alter the sensor material composition (especially silica from the substrates may diffuse to the grain boundaries and hinder the oxygen exchange) and affects the sensor characteristics. To avoid that, the novel room-temperature aerosol deposition technique has been applied. Aerosol deposition is a relatively inexpensive method to obtain dense films directly from the powders without high-temperature firing step [17]. It was investigated for titania as well as for doped SrTiO_3_ (see below). The resistance *vs. p*O_2_-characteristics of the sensors agree well with bulky samples [18]. However, the fact that the ceramic films obtained by aerosol deposition are dense may limit their applicability.

In a more application-oriented research direction, miniaturized sensors are prepared by a sol-gel technique directly on the alumina substrate, on which a resistive heater had been applied beforehand [19]. These sensors were successfully tested and compared with a commercial potentiometric *λ*-probe in real exhausts [20].

## 3. Titanate Sensors for *λ*-Probes

Starting in the early 1990s, several groups tried to circumvent the assumed disadvantages of titania and they investigated doped strontium titanate (=ST) as a material for resistive oxygen sensors. Both thick-film and thin-film sensors were developed. The Härdtl group investigated thick-film sensors. They started with sensors for oxygen determination in process gases [21] and moved later on to the field of automotive exhaust gas sensing. Together with a small enterprise they developed a series of resistive thick-film *λ*-sensors with different sensor characteristics. A short overview on the work of this group can be found in [22].

One focus of their scientific work was to reduce the sensor response times [23]. They tried to detect the *λ*-value of each single cylinder. Therefore, they utilized a powerful testing method that uses oscillations of the total pressure [24], which was later also applied by other groups to determine the rate limiting steps of the oxygen exchange kinetics [25] (for details of this pressure modulation method see Section 8). They measured amplitude and phase of the sensor resistance (compared with the total pressure) for a broad frequency spectrum up to 1 kHz and detected the cut-off frequency [24], which is a measure for the sensor response time. Hence, they quantified the influence of sensor morphology and dopant concentration on sensor response times [26].

**Figure 2.**
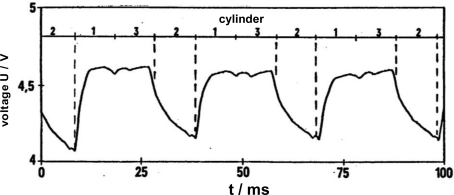
Engine test bench result of a Siemens thin-film sensor [30]. The voltage signal is a result of the changing sensor resistance when operated under different *λ*-values. For further details see text.

At almost the same time, the group of Meixner at Siemens started with sputtered thin-film sensors of doped ST. Several papers were published on this technology and many patents were applied for (e.g., [27–29]). They modified the ST sensor characteristic (*R*(*p*O_2_)-behavior) by sequentially sputtering alternating layers of ST and of metal oxide dopants. After a tempering process, the desired sensors properties appeared [27]. The thin-film sensors with a thickness below 1 μm could reach response times *t*_90_ < 10 ms. Experiments in engine test benches verified the results. In Figure 2, the cylinders were deliberately misadjusted. Cylinder 2 was operated rich (*λ* = 0.9) and cylinder 1 and 3 were adjusted lean (*λ* = 1.1). As can be seen, the sensor is fast enough to detect each cylinder separately.

In spite of this successful manufacturing process, this group also investigated the thick-film technology [31]. Their sample preparation followed conventional routes. In addition, they post-annealed the samples for stability reasons. Whereas the film thickness is the effective diffusion length in a dense film, this characteristic length is given by the grain radius in a porous structure. Therefore, highly porous samples with an average grain size of approx. 2 μm should be as fast as dense thin-films of 1 μm thickness. As shown in Reference [31], porous ST thick-film sensors also respond fast enough to measure the air-to-fuel ratio of each cylinder separately.

## 4. Influence of Dopants on the Sensor Characteristics of Strontium Titanate

**Figure 3.**
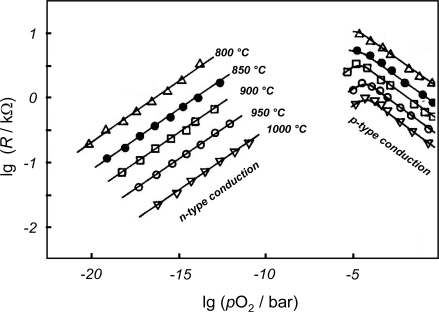
Typical resistance behavior of a ST thick-film sensor (here exemplarily from the Siemens group) that has been post-annealed for 15 h at 1,300 °C. Modified after [31].

The electrical resistance of a typical undoped ST thick-film is shown in Figure 3. Two major drawbacks become obvious: the ambiguity and the temperature dependence of the sensor characteristics. The curve consists of an n-type branch, in which an oxygen uptake (due to an increased oxygen partial pressure, *p*O_2_) reduces the electron concentration and therefore increases the resistivity of the material, and of a p-type region. Here an oxygen uptake goes along with an increased defect electron (hole) concentration, which leads to a decreased resistivity. Such a behavior can be explained very well by defect-chemical means. In particular, ST has been investigated very extensively (for details see [32–34]). Since it is well-known that a slight donor doping causes a right-hand shift of the resistivity curve and a heavy acceptor doping causes a left-hand shift of the sensor characteristics, two different concepts stand to reason to achieve unambiguous sensor characteristics. Depending on the application, each approach has its advantages.

Donor doping leads to a *n*-type conduction and the sensor resistivity increases monotonously with *p*O_2_ in the entire *p*O_2_-range. An exponent *m* = 0.25 can be calculated theoretically. However, since the concentration of oxygen vacancies is low in these materials and since a complete equilibration with the ambient gas atmosphere follows the mass action law of a Schottky-type disorder, which requires diffusion of cations in the lattice, only low response times are predicted by the theory [32]. In contrast to the initial theory, sensor response times of donor doped ST sensors were determined to be in the range of 30 ms at approximately 900 °C [35]. An approach to understand the fast sensor response is given by Meyer and Waser [36]. It is under discussion, whether in general *n*-type conducting oxides can be seen as long-term stable, since one also has to be aware of the Schottky equilibrium, which is usually very slow since it requires diffusion of metal vacancies [32].

In acceptor doped titanates, defect electrons prevail on electrons above an oxygen partial pressure of approximately 10^−10^ bar (depending on temperature and dopant concentration). The acceptors are mainly compensated by oxygen vacancies. A sudden change in the *p*O_2_ of the surrounding atmosphere changes the oxygen vacancy concentration. Incorporation of oxygen ions leads to additional holes and hence the resistance decreases monotonously with increasing *p*O_2_. Elementary calculations give *m* = −0.25. The resulting diffusion process is determined by the high oxygen vacancy mobility, which additionally is enhanced by an ambipolar process [26]. Therefore, sensors should respond very fast to oxygen partial pressure variations. Using the above mentioned measurement technique in the frequency domain, a response time below 4 ms was found for heavily iron doped ST [37].

## 5. Temperature Independent Resistive Oxygen Sensors with *p*-Type Conductors

Another important discovery was that resistive oxygen sensors can be temperature independent. Two systems emerged first: SrTi_1−*x*_Fe*_x_*O_3_ (=STF), and BaFe_1−*y*_Ta*_y_*O_3_ (=BFT). Williams, Tofield, and McGeehin found out that the resistivity of STF becomes temperature independent but remains oxygen dependent for *x* = 0.3...0.4 [38], and Moseley and Williams reported on a temperature independency in BFT for *y* = 0.2...0.3 [39].

**Figure 4.**
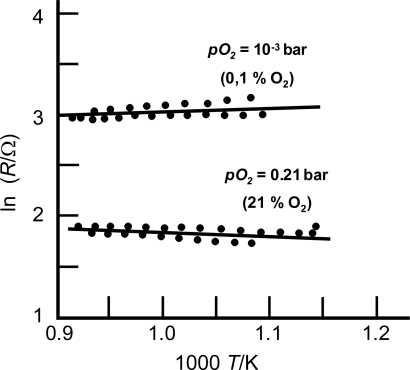
Resistance of a ceramic BaFe_0.8_Ta_0.2_O_3_ sample as a function of temperature. After [39], reprinted with permission from Elsevier.

Figure 4 shows the resistance dependency on temperature of a BaFe_0.8_Ta_0.2_O_3_-ceramic sample for two different oxygen concentrations. The resistance decreases by more than a factor of 3 between 0.1% O_2_ and 21% O_2_, almost invariant with temperature. Using Equation 1, a detailed analysis gives *m* ≈ 0.20 and E_A_ = −0.04 eV for 21% oxygen.

**Figure 5.**
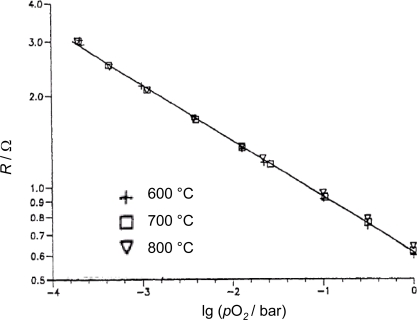
*R*(*p*O_2_)-characteristic of a La_2_CuO_4+δ_ ceramic pellet (after [40]).

Some years later, Blase *et al.* found out that La_2_CuO_4+δ_ (lanthanum cuprate) thick-film sensors also behave temperature independently. In Figure 5, the resistance of a La_2_CuO_4+δ_ ceramic pellet is plotted *vs. p*O_2_ for 600 °C, 700 °C, and 800 °C [40].

Obviously, the temperature dependence can be neglected. An exponent of *m* ≈ 0.2 can be determined from Figure 5. The low measured resistance shows that lanthanum cuprate is a very good electrical conductor. Combined with the temperature independency this offers the chance to heat the sensor film by itself, *i.e.*, to dispense completely with heater and control electronics. These authors also investigated the sensor response times using the frequency domain method. They found cut-off frequencies that correspond to *t*_90_ ≈ 1 ms. The long-term stability under cyclic temperature loading was found to be excellent. However, the main disadvantage of this material type is its instability towards reducing atmospheres and the limited high temperature stability. A comprehensive description of the sensor material and its properties can be found in Reference [41] or in a summary of it [42].

At the same time, Moos *et al.* reinvestigated STF and improved the formulation by adding several dopants in order to extend the *p*O_2_-range of negligible temperature dependence and to shift it to distinct oxygen partial pressures [43,44]. They tried to transfer the properties of the ceramic material to thick-film sensors by following a preparation route described in [31]. However, they found out that sensors prepared that way showed a completely different behavior, as their resistivity increases and becomes temperature dependent. This unexpected effect could be attributed to a reaction between STF sensor film and alumina substrate during firing [45]. Several solutions were suggested and successfully tested. In [45], a strontium spinel intermediate layer between sensitive film and alumina substrate was applied, whereas [46] inserted a bi-directional pressing process between film drying step and firing step. The response time of sensors made of these improved STF compositions was determined to be in the range of a few ms at 900 °C. The aerosol deposition method was also applied. In this case, it could have been clearly demonstrated that the resistance-*vs*-*p*O_2_ characteristics remains unaffected, since without the high-temperature firing step no interdiffusion between substrate and sensor film occurs. Hence, the sensors behave like the ceramic bulk materials [18].

During the past few years, much attention was paid to the explain why the resistivity of STF does not depend on temperature. In an initial approach that considered STF as a doped ST-based material, the activation energy, *E*_a_, of the defect electron (hole) formation was calculated by Equation 2 [35]:

(2) Ea=Egap−12ΔHred

The band gap, *E*_gap_, decreases by increasing the iron content *x* of STF, and since the reduction enthalpy Δ*H*_red_ is considered as constant, at a distinct iron constant (somewhere between *x* = 0.3 and 0.4), *E*_a_ becomes zero. However, this very simplified model is only valid if one considers STF as a slightly doped ST material and if one neglects the strong temperature dependency of the mobile defect electrons. Typically, the mobility of the charge carriers is limited by phonon scattering and decreases with increasing temperature, described usually by a power law with an exponent in the order of 1.5. However, the conduction of electrons or defect electrons occurs via localized states at high iron concentrations and its temperature dependency may vary. In Reference [47], Rothschild *et al.*, found a *T*^−4.5^ dependence. Therefore, they addressed the temperature independent resistivity behavior of STF to the strong temperature dependent hole mobility in conjunction with the decreasing band gap stemming from additional bands of Fe^3+^/Fe^4+^, lying in the bandgap of pure SrTiO_3_. The decrease in band gap is explained by broadening of the 3d band. This band lies above the valance band of SrTiO_3_ and therefore reduces the band gap of the material [48], leading to a kind of impurity band rather than to discrete states. With this physical picture, it is possible to explain the temperature independent resistance of STF in terms of the hole mobility and the Fermi energy level [48]. The latter is, of course, a function of band gap and of oxygen partial pressure. For a temperature dependent *p*-type semiconductor, the Fermi level has to be close to the valence band, so that the thermal generation of the charge carriers is negligibly low, and in addition, the temperature dependence of the mobility has to be quite large to compensate the thermal generation of the charge carriers.

Reference [48] also proposes a defect-chemical model of STF. The often used assumption that iron dopes the ST material is according to [48] not correct. Rather, the system should be seen as a continuous solid solution between strontium titanate ST (SrTiO_3_) and strontium ferrite (SrFeO_3_). Of course, many oxygen vacancies are introduced by the huge amount of Fe^3+^ in STF with *x* = 0.3 ... 0.4, compared to pure ST, however Fe^3+^ is not a lattice defect but an integral component of the system. With this model it is possible to describe the temperature independent resistivity as a function of the oxygen partial pressure. At lower temperature, e.g., *T* < 350 K, polaron conduction is proposed to be the main conduction mechanism [49].

All these sensors are exposed to the harsh environment of internal combustion engines or combustion process flues. Besides high temperatures, varying flow rates, varying oxygen concentrations, water droplets during heating-up, soot particles, *etc.*, sulfur dioxide is one of the most critical components, at least in the automotive exhaust. As was demonstrated for several perovskite materials, very small concentrations of a few ppm sulfur oxide suffice to deteriorate sensors and catalysts [50,51]. A deeper understanding of the poisoning process could lead to the development of a material which is less prone for sulfur poisoning. With special respect to STF, a model was proposed, which describes the poisoning mechanism based on electrical measurements of STF-sensors and bulk material samples in sulfuric oxide containing atmospheres [52]. STF is almost immediately poisoned by sulfuric oxides due to the enormous stability of SrSO_4_, which has been determined in the poisoned gas sensors by X-ray-diffraction. If the poisoning substance is sulfur dioxide (SO_2_), an oxidation of the sulfur from S^IV^ to S^VI^ has to be taken into account. The adsorbed or chemisorbed SO_2_-molecule is able to capture holes from the conduction band of STF, therefore the resistance rises immediately after the first contact with SO_2_. It seems that the subsequent oxidation to sulfur trioxide or sulfate is only possible at temperature above 700 °C [52]. According to [52], at lower temperatures (e.g., *T* < 600 °C) only the interfaces of the ceramic grains to the ambience are sulfur oxide covered, and hence, especially for sensors with dense films or sensors with porous but large grain-sized films, the impact of SO_2_ is markedly lower. In contrast, the response time of such samples at these low temperatures may be not sufficient.

Therefore, new paths were adopted to solve the sulfur problem. The most straightforward idea was to simply keep sulfur dioxide away from the sensor, and to this end sulfur adsorbers were added on the sensor films, usually separated by a porous insulating layer. This concept was firstly claimed by the Siemens research group. To avoid sensor deterioration due to exposition of their ST thin-film sensors to SO_2_, they suggested covering the sensitive films with a porous thick-film titanate layer [53]. Later, they improved their adsorber concept and added an electrically insulating layer between adsorber and sensor [54]. Carbonate based adsorbers were suggested by [55]. Accelerated sulfur aging tests in an extremely sulfur dioxide enriched synthetic exhaust gas were conducted (40 ppm SO_2_ in the gas). Up to almost 500 hours no sensor deterioration could be observed in the synthetic exhaust [55]. 40 ppm SO_2_ in the gas correspond to approximately 8,000 mg sulfur compounds in 1 kg fuel. For comparison: the European Union has mandated the reduction of sulfur levels in gasoline and diesel fuels to 10 mg/kg sulfur by 2009 (directive 2003/17/EC).

**Figure 6.**
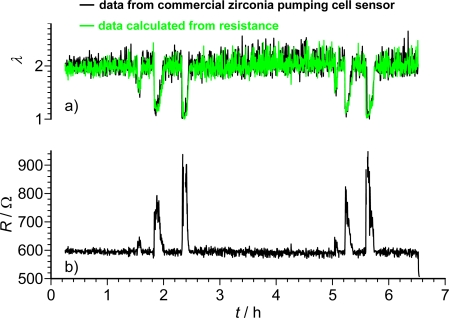
**(a)** Part of a real exhaust test that compared a resistive and a zirconia pumping cell oxygen sensor. **(b)** Measured resistance of the STF sensor. Note: the good agreement between both types of sensors. After [45], reprinted with permission from Elsevier.

A sequence of a long-term test in real exhaust gas is shown in Figure 6. A commercial zirconia pumping cell oxygen sensor was compared with a carbonate adsorber covered doped STF-sensor. The normalized air-to-fuel ratio, *λ*, was kept constant mostly at *λ* ≈ 2. Every now and then, *λ* was reduced. The STF sensor resistance was measured [Figure 6(b)] and *λ* was calculated. This calculated *λ* is plotted together with the measured *λ* of the commercial pumping cell sensor [Figure 6(a)]. Obviously, the resistive oxygen sensor can compete with the zirconia pumping cell.

Very recently, Neri *et al.* reinvestigated the STF system. In contrast to the typical solid-state reaction (mixed oxide technique) used by other authors to prepare STF powders, they applied a self-propagating high-temperature synthesis (SHS) method, in which the powder is formed during a combustion process at temperatures over 2,000 °C [56]. A high energy ball-milling treatment followed. The screen-printed porous sensors consisted of primary grains down to 40 nm diameter [57]. The results are scientifically interesting, since these authors found a temperature independent behavior at an iron content of *x* = 0.6, which is in strong contrast to the values of *x* = 0.3 ... 0.4 found by previous groups on powders prepared in the conventional route, see above and [18,35,38,45]. The question remains open, whether due to the small crystallite size the defect chemistry of the nano-STF materials has to be carefully reinvestigated with respect to the grain size effects, or whether high-energy ball milling and/or propagating high-temperature synthesis affect the materials properties themselves. Nevertheless, it is noteworthy to mention that sensor devices made out of this iron-rich composition have been very successfully tested in both engine test bench and automobile tailpipe on the road [58].

**Figure 7.**
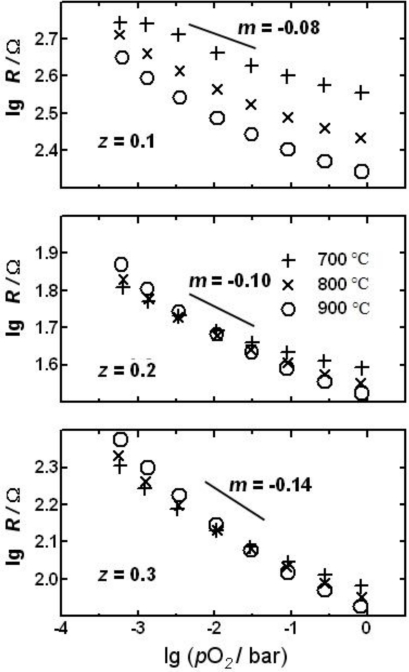
Characteristics of thick-film sensors of LaFe_1−*z*_Cu*_z_*O_3_ [59].

Since it was found out that materials containing earth alkalines are affected by sulfur dioxide in the exhaust, earth alkaline-free formulations were investigated. A temperature independent formulation that came up recently is LaFe_1−*z*_Cu*_z_*O_3_ (=LFC). In Figure 7, the resistance *vs.* oxygen partial pressure behavior of LFC-sensors is plotted for sensors made of materials with three different compositions (*z* = 0.1, *z* = 0.2, and *z* = 0.3).

As can be seen, the sensor characteristics converge with increasing copper content, *i.e.*, the sensor resistance becomes more and more temperature independent. In addition, it can be seen that the oxygen sensitivity (slope *m*) increases with increasing copper content. A good compromise with respect to temperature independency and oxygen sensitivity are LFC-materials with *z* = 0.2...0.3, because they are nearly temperature independent for oxygen concentrations between 1% and 10% (*λ* ≈ 1.05...2). In contrast to STF, the slope is a function of the oxygen partial pressure. With respect to temperature independency, the “novel” material can compete with the “old” STF. However, it should be stressed that STF sensors have *m*-values between 0.2 and 0.24, leading to a remarkable better oxygen sensitivity even compared to the best formulation of the LFC system (*z* = 0.3), where the *m*-value is only 0.14.

In the XRD-patterns, small La_2_CuO_4+δ_-lines appear for *z* = 0.3 and above, indicating to a solubility limit for copper in the perovskite LaFeO_3_ host. In Reference [60] this was studied in detail. In particular, one obtains temperature-independent devices at low copper contents, thus combining the advantages of pure lanthanum cuprate with the sulfur stability of the lanthanum ferrate. In order to correlate sensor characteristics and material composition, a model that is based on the so-called generalized effective media theory (GEMT) is proposed. The GEMT is a useful tool for describing two-phase composites [61]. It allowed even to predict the actual sensor characteristics determined by the experiments.

## 6. Resistive Oxygen Sensors Based on *n*-Type Conducting Metal Oxides

Similar to titania, gallium oxide (Ga_2_O_3_) is another intrinsically *n*-type conducting metal oxide that has been investigated by several groups. Gallium oxide has only one stable form (the monoclinic β-form) with a high melting point of over 1,700 °C. These properties make it a candidate for high temperature oxygen sensors for harsh environments. Fleischer *et al.* were the first to study Ga_2_O_3_ thin-films for gas sensor applications. Their sputtered films had a thickness of several μm and showed a typical resistivity dependence according to Equation 1 with *m* ≈ 0.25 [62]. Already the initial tests revealed a good long-term stability [63]. Due to its bijective characteristics it has been investigated in real engine exhaust with promising behavior [64]. However, the high thermal activation energy of the conductivity of about 2 eV make an application without exact temperature control difficult. Strong anomalies in the *R*(*p*O_2_) behavior at very low *p*O_2_ suggest a phase transition at strongly reducing conditions (high *T*, low *p*O_2_) [65]. Furthermore, it was found out that the sensor response of Ga_2_O_3_ towards reducing components like ethanol or propane cannot be neglected in comparison with the oxygen response even at high temperature of 800 °C. By adding additional catalytically active layers, this cross-effect can be partly suppressed [66]. Several other research groups investigated Ga_2_O_3_ for resistive oxygen purposes. They investigated the influence of sputtering parameters [67] or the effect of dopants [68] on the sensing properties. Also, chemical solution deposited films were prepared on silicon substrates [69]. However, one has to state that up to now, Ga_2_O_3_ has not been used as an oxygen sensor material in commercial applications. Besides patent issues, one reason for that may be the strong temperature dependence of resistivity and the sensor response time in the range of several seconds. The cross-sensitivity to reducing components, which also negatively affects the applicability as an oxygen sensor, however, is the key issue for applications as a selective gas sensor, making them comparable or even better than the classical Taguchi gas sensor [70].

Cerium oxide (ceria) is another *n*-type conducting metal oxide. It is well-known as an oxygen storage material for exhaust gas three way catalysts [71,72]. It can change its oxidation state from Ce^3+^ (Ce_2_O_3_) to Ce^4+^ (CeO_2_) and, therefore, it can be applied for oxygen storage purposes. Since the resistivity of ceria depends strongly on the oxygen partial pressure, ceria can also be used for oxygen sensing purposes. Its *p*O_2_-dependency has been extensively studied and the behavior can be explained by defect chemical means [73,74].

By adding zirconia to ceria, a ceria-zirconia mixed oxide can be formed. This improves both the thermal stability and the oxygen storage capacity, which has been shown to depend on the Ce/Zr-ratio [75]. Also the influence of further dopants like La, Nd [76], and Pr [77] for a higher oxygen storage capacity has been examined. By reducing the particle size of the ceria or ceria-zirconia particles, the oxygen storage effect can be enhanced as well. Kosacki *et al.* reduced the resistivity by forming nanocrystalline ceria. This reduces the enthalpy of oxygen vacancy formation [78]. Tschöpe developed a space charge model based on the defect chemical explanations, that can be applied for mixed ionic/electronic conductors [79]. It explained the effect of the grain size on the resistivity and its *p*O_2_-dependence.

These findings were used for ceria-based resistive oxygen sensors. Beie and Gnörich [80] investigated the oxygen gas sensing properties of thick-films and thin-films of ceria between 700 °C and 1,100 °C, since in that temperature range ceria exhibits a high diffusion coefficient for oxygen vacancies. In their work, thick-films were formed by screen-printing utilizing a paste including cerium oxide powders with an average particle size in the order of several microns. Therefore, the response time of those sensors was in the range of some minutes. They also prepared thin film sensors and claimed that these devices responded within 10 ms. However, no data were shown. Chemical solution deposited ceria thin film sensors with nanocrystalline grains with a size of about 20 nm were prepared by Jasinski *et al.* [81]. The authors found a slope of *m* ≈ 0.25 between 550 °C and 750 °C and an *n*-type conduction in the *p*O_2_-range between 10^−4^ bar and 1 bar. They found response times in the order of several ten seconds, but claimed that the slow response is limited by the slow gas exchange in the sensor chamber. Unfortunately, it seems that both NO_2_ and SO_2_ traces affected the sensor response permanently [82].

A new precipitation method using carbon powder [82] has been developed as a synthesis method for fine nanosized powders. This new precipitation method is suitable to produce larger synthesis quantities of a powder than other methods. Izu *et al.* reported on resistive type sensors using the fine ceria or ceria-zirconia powder by the new precipitation method [83–85]. The used ceria or ceria-zirconia thick-films were investigated after firing at 1,100 °C by XRD and SEM. The XRD patterns for the ceria/zirconia thick-films showed one single-phased fluorite structure, except for 20 mol% ZrO_2_. Based on the SEM image, the thick-films had a porous structure and an average particle size of 100 nm. The particles observed by SEM may include a few crystallites. The response time of the thick-films with 10 and 20 mol% Zr was 16 ms and 9 ms at 800 °C, respectively. Therefore, it is concluded that the very fast response sensors are due to the nanosized powder [86–88].

There is a linear relationship between the logarithm of the oxygen partial pressure and the logarithm of the sensor resistance at 600 °C and 800 °C in a wide range of oxygen partial pressures. The variable *n* was introduced to describe the sensitivity, with *n* = 1/*m*. Values of *n* (in *R* ∝ *p*O_2_^1/*n*^) in the range of 6.3 and 7.4 were found. Since resistive oxygen sensors based on ceria or ceria-zirconia can measure oxygen partial pressures in a wide air-to-fuel ratio, they cannot only be used for binary *λ* detections around *λ* ≈ 1, but also to measure the air-to-fuel ratio in lean-burn engine exhausts or process gas flues.

A major field of application for oxygen sensors is to monitor the oxygen content in automotive exhaust gases downstream of a three-way catalyst. Recently, ceria-based resistive sensors have been applied to directly monitor the oxygen loading of three-way catalyst coatings. The catalysts are coated with ceria-zirconia solid solutions as an oxygen storage component. The purpose of the oxygen measurement in the gas phase downstream of the three-way catalyst is to determine the oxygen loading amount. Therefore, oxygen loading models are used that base on the oxygen gas sensor signal [89]. Whether a measurement of the resistance of the ceria-zirconia catalyst coating itself can give an enhanced insight into the catalyst behavior has been investigated in a series of papers [90].

**Figure 8.**
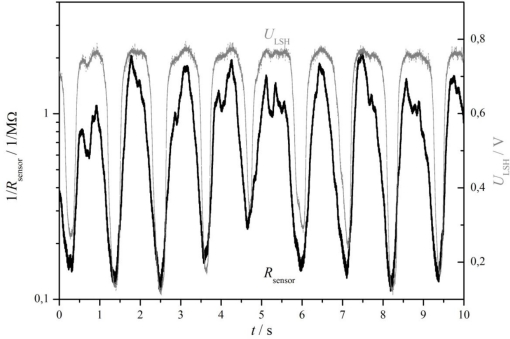
Comparison of the three-way catalyst sensor conductance (1/*R*_sensor_) and the voltage of a binary *λ* probe, *U*_LSH_ (Bosch type LSH) at the engine outlet during typical operation. From [91], reprinted with kind permission from Springer Science+Business Media B.V.

At first it was tested whether a small sensor consisting of a catalyst ceria-zirconia coating applied on an insulating substrate with interdigital electrodes reacts on the oxygen content of the exhaust, *i.e.*, whether it resistance depends on *λ*. A characteristic response of such a sensor is shown in Figure 8 (from [91]). The sensor was installed in the exhaust manifold in front of the three-way catalyst, close to the serial binary zirconia *λ*-probe. The engine operated at constant speed-load conditions. The typical oscillations [1,2] around the stoichiometric point at *λ* ≈ 1 can be clearly seen. They appear in both, the binary zirconia *λ*-probe and the resistive-type catalyst (ceria-zirconia) coating sensor. One gets the impression that the catalyst coating sensor is even faster, showing signal patterns more pronouncedly.

After these successful initial tests, several sensor elements with a catalyst identical coating were mounted into the catalyst device along the flow direction. The normalized sensor signals are a direct measure for the oxygen loading degree at each sensor position. During lean-rich switches, one can nicely see how an oxygen front moves through the catalyst [91]. By summing up the normalized sensor signals on gets one integral parameter that is directly (and almost linearly) correlated with the degree of oxygen loading of the catalyst coating [92].

The oxygen storage capacity of the storage component varies with different ratios of ceria/zirconia [75,93]. Even the presence of alumina as the basis of a TWC coating can enhance the oxygen storage capacity of the ceria-zirconia [94]. Thus, the characteristics of each TWC composition is different and the calculation of the oxygen loading of the TWC has to be adapted to the material used for the catalyst coating. Therefore, the method to measure *in-situ* the resistance of the catalyst coating itself, may help catalyst development, leading to new *in-situ* insights into the catalyst chemistry and may also help to improve catalyst loading models, but for now one cannot expect to bring such systems into a serial development.

Another technique of gauging the electrical resistivity was presented in [95], where microwaves are used to detect the electrical properties of the catalyst material. The propagation of the electromagnetic waves is influenced by the varying resistivity of the oxygen storage component, which leads to a perturbation of a microwave cavity resonator (the exhaust catalyst device). The major advantage of this approach is the contactless measurement technique and thus the simple application in the exhaust gas pipe [96]. Due to the utilized microwave frequency, one may see this system as an unconventional kind of resistive oxygen sensor, since the sensor output is directly correlated with the oxygen dependent resistivity of the ceria-zirconia material. It seems that this principle can be applied for each oxygen-storing three-way catalyst material.

## 7. Temperature Independent *n*-Type Resistive Oxygen Sensors Based on Ceria

As shown above, the temperature dependence of resistivity of *p*-type metal oxide sensing materials can be eliminated by doping. In the case of *n*-type semiconducting materials, however, no mechanism is known to reduce material-intrinsically the temperature dependence of the resistivity of oxygen sensing materials. Therefore, a second material that compensates the temperature dependence is required. On several approaches for temperature compensating materials (TCMs) has been reported in the literature [97–100]. Izu *et al.* have recently suggested the use of oxide ion conductors as long-term stable TCMs suitable for the application in harsh environments [101–103]. In the range of oxygen partial pressures and temperatures, in which the materials exhibit an ionic transference number of 1 (*i.e.*, they are solely ionic conductors; electronic conductivity contributions can be neglected), the total electrical conductivity of an oxide ion conductor is independent on the oxygen partial pressure but temperature dependent similar to an oxide semiconductor. These properties are required for a TCM.

**Figure 9.**
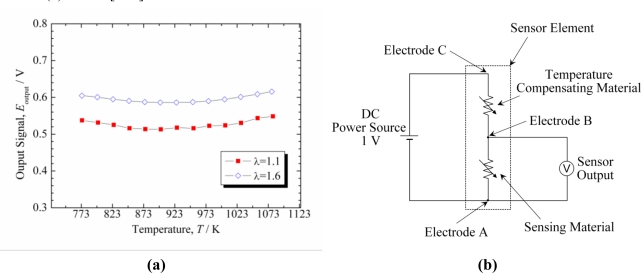
**(a)** Electrical circuit for the sensor element using the temperature compensating material. **(b)** Output signal of the sensor element obtained using the electric circuit shown in (a). From [104].

First, it is explained how to prevent the temperature dependence of an *n*-type oxygen sensor utilizing a TCM in principle. When a constant voltage, *V*, is applied to the serial circuit of TCM and gas sensitive material [Figure 9(a)], the potential difference of the sensing material, *E*_output_, can be expressed as follows:

(3) $$E_{\text{output}}=\frac{r_{o}}{\left(r_{o}+r_{t}\right)}\times V=\frac{r_{o}^{O}\times {pO}_{2}^{1/n}\times \text{exp}\left(\frac{E_{o}}{\mathrm{kT}}\right)}{r_{o}^{O}\times {pO}_{2}^{1/n}\times \text{exp}\left(\frac{E_{o}}{\mathrm{kT}}\right)+r_{t}^{O}\times \;\text{exp}\left(\frac{E_{t}}{\mathrm{kT}}\right)}\times V$$

where *r*_o_^O^ and *r*_t_^O^ are the inherent constant values of sensing material and TCM, respectively, which are independent on oxygen partial pressure and temperature, *p*O_2_ is the oxygen partial pressure, *n* is a parameter indicating oxygen partial pressure dependence (normally 4 to 6), *E*_o_ and *E*_t_ are the activation energies of the conductivity of the sensing material and the TCM, respectively, and *k* is Boltzmann’s constant. If *E*_o_ is the same as *E*_t_, then Equation (3) simplifies to:

(4) $$E_{\text{output}}=\frac{r_{o}^{O}\times {pO}_{2}^{1/n}}{r_{o}^{O}\times {pO}_{2}^{1/n}+r_{t}^{O}}\times V$$

In Equation (4), the temperature dependent term disappears. In other words, the sensor output is independent on temperature.

Next, typical results are shown here. In Reference [104], TCMs were investigated for a resistive oxygen sensor using Ce_0.9_Zr_0.1_O_2_ (CeZr10) as a sensing material for lean-burn engines. The temperature compensating material should have the same temperature dependence of resistance as the sensor material and a resistivity that is independent on the oxygen partial pressure. As a result of an yttria concentration optimization in the TCM CeO_2_-Y_2_O_3_, it has been revealed that CeY50 (Ce_0.5_Y_0.5_O_2−δ_) has the same activation energy of resistance as the oxygen sensitive composition CeZr10 but with a *p*O_2_-independent resistivity. For this composition, XRD analyses revealed no other phase except for fluorite or C-rare earth structures. Therefore, CeY50 was confirmed to be a suitable temperature compensating material. Sensor elements comprised of CeZr10 and CeY50 as sensor and temperature compensating materials were fabricated, and the dependence of the sensor element output on temperature was investigated. The electrodes in the sensor element were optimized to adjust the resistance of CeZr10 to that of CeY50. The output was approximately independent on temperature in a wide range from 500 °C to 800 °C [Figure 9(b)].

## 8. Response Evaluation of Resistive Type Sensors Using the Pressure Modulation Method

It is important to ascertain the factors controlling the mechanisms of the sensor response to varying oxygen concentrations, since this information is essential to reduce the response time of resistive oxygen sensors. An excellent overview on the mechanisms how oxygen is incorporated into metal oxides for the model substance ST is given in Reference [105]. With the pressure modulation method (PMM) the rate-limiting step of the sensor response can be determined, or at least one can distinguish between oxygen volume/bulk diffusion control and surface reaction control [24,25,106–110]. In other words, the PMM can be used as an *in-situ* method for “measuring” oxygen transport parameters in sensor materials, especially the diffusion coefficient, *D*, and the surface reaction parameter, *k*. Further details can be found in [111]. This useful method measures the amplitude of the sensor output, *H*(*f*), for sine wave modulations of the total pressure at constant oxygen percentages at different frequencies, *f*, *i.e.*, the *p*O_2_ is sinusoidally modulated. The PMM can be considered as a kind of spectroscopy. For low frequencies, the sensor response, *H*, follows the *p*O_2_-modulations without delay, *H* remains constant, and sensor output and *p*O_2_ are in phase. With increasing modulation frequency, at a distinct modulation frequency, *f*_0_, the sensor kinetics becomes too slow and the sensor output begins to decrease. From the slope in the double-logarithmic *H*(*f*)-plot (or from the phase response) above *f*_0_, one can determine the rate limiting steps. While a slope of −0.5 is a strong indication for oxygen diffusion control, a slope of −1 is a hint for surface reaction control. The reciprocal value of *f*_0_, τ = *f*_0_^−1^, is a measure for the sensor response time [106].

**Figure 10.**
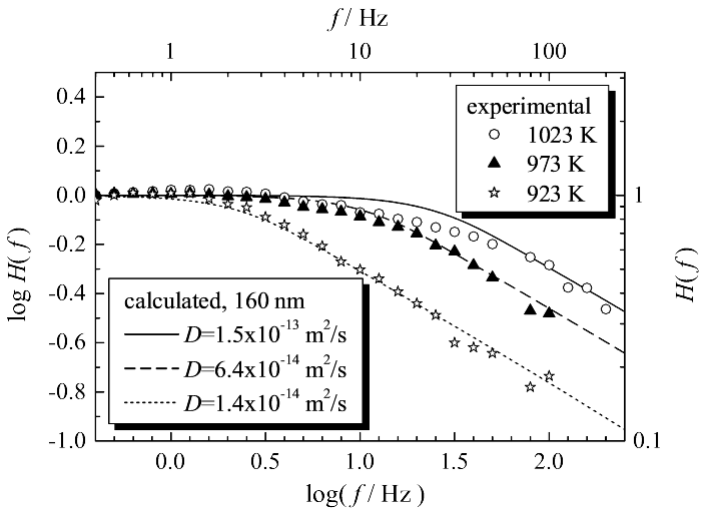
Calculated and measured results for a porous ceria thick film with average particle size of 160 nm obtained by the pressure modulation method (PMM). From [25], reprinted with permission from Elsevier.

Tragut and Härdtl thoroughly investigated ST resistive oxygen sensors by PMM [24,106]. Recently, PMM was applied to determine the rate-limiting steps in ceria-based resistive oxygen sensors. In the experiments, the oxygen partial pressure was changed using a highly efficient servo-valve to change the total air pressure. In Reference [25], by calculated simulation of the PMM, the plot of log *f vs*. log *H*(*f*) in the high *f* region was found to have a slope of approximately −0.5 for both porous thick-film and non-porous thin-films, when the rate-limiting step was diffusion. The calculated simulation also showed that the response time of the porous thick-film was 1/20 that of non-porous thin-film when the grain diameter of the porous thick film was the same as the thickness of the non-porous thin-film. This means that porous thick-films with nanometer particle size are suitable for fast response sensors rather than dense thin-films with nanometer thickness. At 700 °C, τ of the resistive oxygen sensor was found by experiment to be 109 ms (Figure 10). Since the experiment revealed that the slope on the plot of log *f* versus log *H*(*f*) in the high *f* region was approximately −0.5 (Figure 10), it was concluded that the response of the resistive oxygen sensor with an average particle size of about 160 nm was strongly controlled by diffusion at 650 °C to 750 °C. At these temperatures, the diffusion coefficient of oxygen vacancy in porous ceria (*D*_V_) was also calculated.

Sahner *et al.* compared some temperature independent formulations by PMM. STF, with a fine-grained microstructure, responded very fast. Due to the fast oxygen diffusion in these highly acceptor doped perovskites, it is not astonishing that surface reaction was found to be the rate-limiting step up to 900 °C [112]. Interestingly, Argirusis *et al*. discovered that by coating the samples with a thin alkaline earth metal oxide layer (CaO), a significantly enhanced oxygen surface exchange reaction occurs [113]. This recently observed behavior might help to develop sensors with a response time below 1 ms. With temperature-independent LCF, one may reach such fast sensor responses only for very fine-grained nano-materials, since at higher temperature the sensor kinetics becomes oxygen bulk diffusion-controlled [112]. It is noteworthy to mention that the PMM is the only method available at the moment, to determine the sensor response kinetics in the millisecond range. Using a typical “sudden gas concentration step” in a tube furnace or in a small sensor chamber, one usually has to deal with “sensor kinetics” (which in fact are determined by the response time of the gas supply unit) of some seconds, e.g., Reference [114].

## 9. Future Prospect: Utilizing the Thermoelectric Effect

One disadvantage of all resistive sensors is the dependence of the resistance on geometry and morphology of the sensor films. Small losses of the sensing material due to initial cracks in the sensor film or even a densification due to sintering processes affect the sensor resistances and modify the sensor characteristics, making an in-operation recalibration necessary. Whereas the geometry dependence is crucial for reproducible manufacturing, the first issues strongly affect long-term stability. Albeit the mobile charge carrier density (electrons or holes) is the path-independent measurand, the material property “conductivity” or “resistivity” can only be determined with the knowledge of geometrical parameters, since the resistance is typically immediately affected if current paths are influenced.

These problems can be overcome if one directly measures the Seebeck coefficient, *Q*, of the sensing materials. It describes the position of the Fermi-level in the band scheme and is correlated with the electronic charge carrier densities, *n* or *p*, which depend on the oxygen partial pressure as shown above. For example, in *p*-type semiconducting oxides, Equation 5 holds [115]:

(5) $$Q=+\frac{k}{e}\cdot \left(\text{ln}\frac{N_{V}}{p}+A_{h}\right)$$

**Figure 11.**
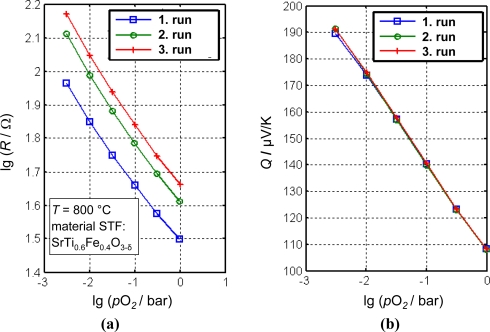
**(a)** Resistance, *R*, and **(b)** thermopower (Seebeck coefficient), *Q*, of a porous STF specimen when exposed to different oxygen partial pressures.

In Equation 5,*k* stands for Boltzmann’s constant, *e* for the electron charge, *N*_V_ for the effective density of states in the valence band, and *A*_h_ is a scattering mechanism-dependent transport coefficient for defect electrons. Unlike the resistance, *Q* is independent on the geometry of the gas sensitive materials. Despite the relationship between Seebeck coefficient and the conductivity of metal oxides was evaluated very early by Jonker [115], only a few works are known using this principle for oxygen gas sensing purposes (see below). The advantage of so-called direct thermoelectric sensors becomes immediately clear from Figure 11.

In Figure 11, the thermopower, *Q*, and the resistance, *R*, of a small porous brick-shaped STF ceramic were measured simultaneously (details in [116]). For each run, the samples were kept at 700 °C, 800 °C, and 900 °C for 7 h. Within these 7 h, the oxygen partial pressure was varied stepwise and the final values of *R* and *Q* were plotted. As can be seen, the resistance characteristics of the material shifts from run to run, presumably due to a sintering process of the sample. This shift amounts to an error of approximately one decade in *p*O_2_. The thermopower, however, remains constant. This experiment clearly highlights the advantage of the so-called direct thermoelectric sensors.

The disadvantages of such sensors are that a basic sensor setup comprises at least a heater to bring the whole device to operation temperature, insulating layers, and a (separate) heater providing a modulated temperature gradient Δ*T* over the gas sensitive film. Furthermore, the measured thermovoltages of the materials are low, and the slopes of pure *n*-type or pure *p*-type semiconductors are mostly below 50 μV/K per decade *p*O_2_ [117].

Suitable materials for direct thermoelectric gas sensors are semiconducting oxides, if oxygen diffusion is fast enough, and if the electronic charge carrier density is affected significantly by *p*O_2_-fluctuations. In other words, the same materials that are known for resistive oxygen sensing are of interest as direct thermoelectric oxygen sensors. Some examples for realized sensor devices to measure oxygen in the pure *p*-type regime are shown in [118]. For STF sensors, slopes of only 52 μV/K per decade *p*O_2_ were found out. A by far higher sensitivity can be obtained, if one dopes the materials in a way that shifts the S-shaped *Q*(*p*O_2_)-curve (see [119]) into the intrinsic regime, in which donors and acceptors compensate themselves. In this region, slight *p*O_2_-fluctuations suffice to alter the value of *Q*. Sensors made of an intrinsic oxide semiconductor showing an almost linear slope in the *Q*(*p*O_2_)-plot of 186 μV/K per decade *p*O_2_ were obtained [120].

**Figure 12.**
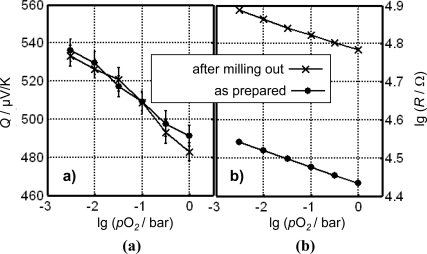
**(a)** Thermopower and **(b)** resistance of a thermoelectric gas sensor before and after milling out the sensitive layer as indicated. From [122].

The huge advantage of the direct thermoelectric gas sensors is also impressively demonstrated in Figure 12, here for Cr_2_O_3_ as the gas sensitive material at 700 °C. A sensor was prepared as outlined above (similar to [121]). The sensor was measured twice under the same conditions. In between, a part of the sensitive film was deliberately milled out. Figure 12 compares resistance and thermopower before and after milling out a part of the gas sensitive Cr_2_O_3_-layer. While the resistance increased by almost half a decade after milling out, the thermopower remained the same.

Although the mechanisms leading to the charge carrier concentration changes are the same, the effect of temperature is different and up to now, no temperature independent material has been found. Interestingly, this brings us back where we started. If one uses ion-conducting YSZ as a thermoelectric sensor material, one finds a temperature independent behavior [123].

For a complete sensor, it has to be taken into account that the measurement of the thermopower is costlier compared to the resistive measurement, e.g., two temperatures (or one temperature difference) and the thermovoltage have to be measured. Additionally, the internal sample resistance has to be low enough and the data evaluation algorithm needs to be more complicated [124]. If these boundary conditions are met, the measurement of the thermopower leads to the described advantages and the here-presented resistive materials can also be used as direct thermoelectric gas sensors.

## 10. Conclusions

Despite the fact that research on resistive oxygen sensors has been conducted for over 40 years, new approaches still appear. In the beginning, research largely focused on materials screening, mostly on *n*-type semiconducting metal oxides due to the bijective *R*(*p*O_2_) characteristics. Later, several attempts were undertaken to address the issue of temperature dependence (mostly utilizing *p*-type semiconducting oxides), sulfur resistance and resistance towards reducing atmospheres. For classical materials, recent approaches deal with manufacturing technologies, whereas recent applications in catalysis deal with the *in-situ* determination of the catalyst status. In order to improve long-term stability in critical exhausts, adsorbers or new material formulations are under study. In another recent approach, the thermoelectric power is utilized as a geometry-independent and abrasion-resistant measurand instead of the voltage drop as a result of an impressed current. From a scientific point of view, the pressure modulation method is worth to be mentioned. On the one hand, it allows one to determine sensor response times, even if they are in the millisecond range, and on the other hand, it is a powerful tool to determine the rate-limiting steps that determine the sensor kinetics.

## References

[1] Twigg MV (2007). Progress and future challenges in controlling automotive exhaust gas emissions. Appl. Catal. B Environ.

[2] Riegel J, Neumann H, Wiedenmann H-M (2002). Exhaust gas sensors for automotive emission control. Solid State Ionics.

[3] Baunach T, Schänzlin K, Diehl L (2006). Sauberes Abgas durch Keramiksensoren. Physik J.

[4] Gibbons EF, Meitzler AH, Foote LR, Zacmanidis PJ, Beaudoin GL (1975). Automotive exhaust sensors using titania ceramic. SAE Tech Paper.

[5] Smyth DM (2000). Titanium dioxide. The Defect Chemistry of Metal Oxides.

[6] Tien TY, Stadler HL, Gibbons EF, Zacmanidis PJ (1975). TiO_2_ as an air-to-fuel ratio sensor for automobile exhausts. Ceram. Bull.

[7] Kaiser WJ, Logothetis EM (1983). Exhaust gas oxygen sensors based on TiO_2_. SAE Tech Paper.

[8] Howarth DS, Micheli AL (1984). A simple titania thick film exhaust gas oxygen sensor. SAE Tech Paper.

[9] Takami A, Matsuura T, Miyata S, Furusaki K, Watanabe Y (1987). Effect of precious metal catalyst on TiO_2_ thick film HEGO sensor with multi-layer alumina substrate. SAE Tech Paper.

[10] Takami A (1988). Development of titania heated exhaust-gas oxygen sensor. Ceram. Bull.

[11] Takeuchi T (1988). Oxygen sensors. Sens. Actuat. B Chem.

[12] Radecka M, Zakrzewska K, Rekas M (1998). SnO_2_-TiO_2_ solid solutions for gas sensors. Sens. Actuat. B Chem.

[13] Haghighat F, Khodadadi A, Mortazavi Y (2008). Temperature-independent ceria- and Pt-doped nano-size TiO_2_ oxygen lambda sensor using Pt/SiO_2_ catalytic filter. Sens. Actuat. B Chem.

[14] Francioso L, Presicce DS, Siciliano P, Ficarella A (2007). Combustion conditions discrimination properties of Pt-doped TiO_2_ thin film oxygen sensor. Sens. Actuat. B Chem.

[15] Zhang M, Yuan Z, Song J, Zheng C (2010). Improvement and mechanism for the fast response of a Pt/TiO_2_ gas sensor. Sens. Actuat. B Chem.

[16] Trimboli J, Dutta PK (2004). Oxidation chemistry and electrical activity of Pt on titania: Development of a novel zeolite-filter hydrocarbon sensor. Sens. Actuat. B Chem.

[17] Akedo J (2006). Aerosol deposition of ceramic thick films at room temperature: Densification mechanism of ceramic layers. J. Am. Ceram. Soc.

[18] Sahner K, Kaspar M, Moos R (2009). Assessment of the aerosol deposition method for preparing metal oxide gas sensors at room temperature. Sens. Actuat. B Chem.

[19] Francioso L, Presicce DS, Epifani M, Siciliano P, Ficarella A (2005). Response evaluation of TiO_2_ sensor to flue gas on spark ignition engine and in controlled environment. Sens. Actuat. B Chem.

[20] Presicce DS, Francioso L, Epifani M, Siciliano P, Ficarella A (2005). Temperature and doping effects on performance of titania thin film lambda probe. Sens Actuat B Chem.

[21] Schönauer U (1989). Thick-film oxygen gas sensors based on ceramic semiconductors. Technisches Messen.

[22] Häfele E, Schönauer U, Seeger W (1993). Sensorsysteme für Low-Emission-Fahrzeuge mit Katalysator-Überwachung (in German). Automobiltechnische Zeitschrift.

[23] Schönauer U (1991). Response times of resistive thick-film oxygen sensors. Sens. Actuat. B Chem.

[24] Tragut C, Härdtl KH (1991). Kinetic behavior of resistive oxygen sensors. Sens. Actuat. B Chem.

[25] Izu N, Shin W, Matsubara I, Murayama N (2006). Evaluation of response characteristics of resistive oxygen sensors based on porous cerium oxide thick film using pressure modulation method. Sens. Actuat. B Chem.

[26] Müller A, Härdtl KH (1989). Ambipolar diffusion phenomena in BaTiO_3_ and SrTiO_3_. Appl. Phys. A.

[27] Gerblinger J, Meixner H (1991). Method for Manufacturing a Fast Oxygen Sensor. European Patent 0,498,916.

[28] Gerblinger J, Meixner H (1990). Electrical conductivity of sputtered films of strontium titanate. J. Appl. Phys.

[29] Meixner H, Gerblinger J, Lampe U, Fleischer M (1995). Thin-film gas sensors based on semiconducting metal oxides. Sens. Actuat. B Chem.

[30] Gerblinger J (1991). Sauerstoffsensoren auf der Basis gesputterter Strontiumtitanat-Schichten (in German).

[31] Gerblinger J, Hauser M, Meixner H (1995). Electric and kinetic properties of screen-printed strontium titanate films at high temperatures. J. Am. Ceram. Soc.

[32] Moos R, Härdtl KH (1997). Defect chemistry of donor doped and undoped strontium titanate ceramics between 1,000 °C and 1,400 °C. J. Am. Ceram. Soc.

[33] Smyth DM (2000). Barium titanate. The Defect Chemistry of Metal Oxides.

[34] Tuller HL, Buchanan RC (1991). Highly conductive ceramics. Ceramic Materials for Electronics.

[35] Menesklou W, Schreiner H, Härdtl KH, Ivers-Tiffée E (1999). High temperature oxygen sensors based on doped SrTiO_3_. Sens. Actuat. B Chem.

[36] Meyer R, Waser R (2004). Resistive donor-doped SrTiO_3_ sensors: I, basic model for a fast sensor response. Sens. Actuat. B Chem.

[37] Ivers-Tiffée E, Härdtl KH, Menesklou W, Riegel J (2001). Principles of solid state oxygen sensors for lean combustion gas control. Electrochimica Acta.

[38] Williams DE, Tofield BC, McGeehin P (1982). Oxygen Sensors. European Patent 0,062,994.

[39] Moseley P, Williams DE (1989). Gas sensors based on oxides of early transition metals. Polyhedron.

[40] Blase R, Härdtl KH, Schönauer U (1997). Oxygen Sensor Based on Non-Doped Cuprate. US Patent 5,792,666.

[41] Blase R (1994). Temperaturunabhängige Sauerstoffsensoren mit kurzer Einstellzeit auf der Basis von La_2_CuO_4+δ_-Dickschichten (in German).

[42] Blase R, Härdtl KH (1996). Schneller Sauerstoffsensor zur Regelung von Verbrennungsvorgängen (in German).

[43] Moos R, Menesklou W, Schreiner H-J, Härdtl KH (1997). Oxygen Sensor. US Patent 6,319,429.

[44] Moos R, Menesklou W, Schreiner HJ, Härdtl KH (2000). Materials for temperature independent resistive oxygen sensors for combustion exhaust gas control. Sens. Actuat. B Chem.

[45] Moos R, Rettig F, Hürland A, Plog C (2003). Temperature-independent resistive oxygen exhaust gas sensors for lean-burn engines in thick-film technology. Sens. Actuat. B Chem.

[46] Schreiner H-J, Menesklou W, Härdtl KH (1999). Haftfeste Dickschicht-Sauerstoffsensoren für Magermotoren (in German). German Patent DE 19,927,725.

[47] Rothschild A, Litzelman SJ, Tuller HL, Menesklou W, Schneider T, Ivers-Tiffée E (2005). Temperature-independent resistive oxygen sensors based on SrFe_1-x_Ti_x_O_3-δ_ solid solutions. Sens. Actuat. B Chem.

[48] Rothschild A, Menesklou W, Tuller HL, Ivers-Tiffée E (2006). Electronic structure, defect chemistry, and transport properties of SrTi_1-x_Fe_x_O_3-y_ solid solutions. Chem. Mater.

[49] Zhou HD, Goodenough JB (2004). Polaron morphologies in SrFe_1-x_Ti_x_O_3-δ_. J. Solid State Chem.

[50] Wan L, Tejuca LG, Fierro JLG (1993). Poisoning of perovskite oxides by sulfur dioxide. Properties and Applications of Perovskite-Type Oxides.

[51] Schulte T, Waser R, Römer EWJ, Bouwmeester HJM, Nigge U, Wiemhöfer H-D (2001). Development of oxygen-permeable membranes for NO_x_-sensors. J. Eur. Ceram. Soc.

[52] Rettig F, Moos R, Plog C (2004). Poisoning of temperature independent resistive oxygen sensors by sulfur dioxide. J. Electroceram.

[53] Gerblinger J, Lampe U, Meixner H (1993). Gas Sensor. European Patent EP 0,656,538.

[54] Meixner H, Kornely S, Hahn D, Leiderer H, Lemire B, Hacker B (1995). Gas Sensor. U.S. Patent 6,101,865.

[55] Rettig F, Moos R, Plog C (2003). Sulfur adsorber for thick-film exhaust gas sensors. Sens. Actuat. B Chem.

[56] Sanson A, Mercadelli E, Roncari E, Licheri R, Orrù R, Cao G, Merlone-Borla E, Marzorati D, Bonavita A, Micali G, Neri G (2010). Influence of processing parameters on the electrical response of screen printed SrFe_0.6_Ti_0.4_O_3-δ_ thick films. Ceram. Int.

[57] Neri G, Bonavita A, Micali G, Rizzo G, Licheri R, Orrù R, Cao G (2007). Resistive λ-sensors based on ball milled Fe-doped SrTiO_3_ nanopowders obtained by self-propagating high-temperature synthesis (SHS). Sens. Actuat. B Chem.

[58] Neri G, Micali G, Bonavita A, Licheri R, Orrù R, Cao G, Marzorati D, Merlone Borla E, Roncari E, Sanson A (2008). FeSrTiO_3_-based resistive oxygen sensors for application in diesel engines. Sens. Actuat. B Chem.

[59] Moos R, Rettig F (2003). Resistiver Sauerstoffsensor (in German). German Patent DE 10114645 C1.

[60] Sahner K, Straub J, Moos R (2006). Cuprate-ferrate compositions for temperature independent resistive oxygen sensors. J. Electroceram.

[61] McLachlan DS (1987). An equation for the conductivity of binary mixtures with anisotropic grain structures. J. Phys. C Solid State Phys.

[62] Fleischer M, Meixner H (1991). Gallium oxide thin films: A new material for high-temperature oxygen sensors. Sens. Actuat. B Chem.

[63] Fleischer M, Meixner H (1991). Oxygen sensing with long-term stable Ga_2_O_3_ thin films. Sens. Actuat. B Chem.

[64] Lampe U, Fleischer M, Meixner H (1994). Lambda measurement with Ga_2_O_3_. Sens. Actuat. B Chem.

[65] Fleischer M, Höllbauer L, Born E, Meixner H (1997). Evidence for a phase transition of β-Gallium oxide at very low oxygen pressures. J. Am. Ceram. Soc.

[66] Schwebel T, Fleischer M, Meixner H (2000). A selective, temperature compensated O_2_ sensor based on Ga_2_O_3_ thin films. Sens. Actuat. B Chem.

[67] Ogita M, Higo K, Nakanishi Y, Hatanaka Y (2001). Ga_2_O_3_ thin film for oxygen sensor at high temperature. Appl Surf Sci.

[68] Li Y, Trinchi A, Wlodarski W, Galatsis K, Kalantar-zadeh K (2003). Investigation of the oxygen gas sensing performance of Ga_2_O_3_ thin films with different dopants, *Sens*. Actuat. B Chem.

[69] Bartic M, Ogita M, Isai M, Baban C-L, Suzuki H (2007). Oxygen sensing properties at high temperatures of β-Ga_2_O_3_ thin films deposited by the chemical solution deposition method. J. Appl. Phys.

[70] Hoefer U, Frank J, Fleischer M (2001). High temperature Ga_2_O_3_-gas sensors and SnO_2_-gas sensors: A comparison. Sens. Actuat. B Chem.

[71] Yao HC, Yu Yao YF (1984). Ceria in automotive exhaust catalysts. J. Catal.

[72] Kašpar J, Fornasiero P, Graziani M (1999). Use of CeO_2_-based oxides in the three-way catalysis. Catal. Today.

[73] Tuller HL, Nowick AS (1979). Defect structure and electrical properties of nonstoichiometric CeO_2_ single crystals. J. Electrochem. Soc.

[74] Kim S, Maier J (2002). On the conductivity mechanism of nanocrystalline ceria. J. Electrochem. Soc.

[75] Suda A, Ukyo Y, Yamamura K, Sobukawa H, Sasaki T, Nagai Y, Tanabe T, Sugiura M (2004). Effect of ordered arrangement of Ce and Zr ions on oxygen storage capacity of ceria-zirconia solid solutions. J. Ceram. Soc. Jpn.

[76] Nakatani T, Wakita T, Wakasugi T, Ota R (2004). Oxygen store and release of CeO_2_-ZrO_2_-MO_1.5_ (M = La, Nd) powders prepared by co-precipitation method. J. Ceram. Soc. Jpn.

[77] Stefanik TS, Tuller HL (2001). Ceria-based gas sensors. J. Eur. Ceram. Soc.

[78] Kosacki I, Suzuki T, Petrovsky V, Anderson HU (2000). Electrical conductivity of nanocrystalline ceria and zirconia thin films. Solid State Ionics.

[79] Tschöpe A (2001). Grain size-dependent electrical conductivity of polycrystalline cerium oxide II: Space charge model. Solid State Ionics.

[80] Beie H-J, Gnörich A (1991). Oxygen gas sensors based on CeO_2_ thick and thin films. Sens. Actuat. B Chem.

[81] Jasinski P, Suzuki T, Anderson HU (2003). Nanocrystalline undoped ceria oxygen sensor. Sens. Actuat. B Chem.

[82] Murayama N, Izu N, Shin W, Matsubara I (2005). Preparation of SnO_2_ nanosized powder by precipitation method with nano-mixing of carbon powder. J. Ceram. Soc. Jpn.

[83] Izu N, Shin W, Matsubara I, Murayama N (2004). Resistive oxygen gas sensors using ceria-zirconia thick films. J. Ceram. Soc. Jpn.

[84] Izu N, Oh-hori N, Shin W, Matsubara I, Murayama N, Itou M (2008). Response of resistive oxygen sensors using Ce_1-x_Zr_x_O_2_ (x = 0.05, 0.10) thick films in propane combustion gas. Sens. Actuat. B Chem.

[85] Izu N, Shin W, Matsubara I, Itoh T, Nishibori M, Murayama N (2010). Pt catalytic effects on a resistive oxygen sensor using Ce_0.9_Zr_0.1_O_2_ thick film in rich conditions. J. Ceram. Soc. Jpn.

[86] Izu N, Oh-hori N, Itou M, Shin W, Matsubara I, Murayama N (2005). Resistive oxygen gas sensors based on Ce_1-x_Zr_x_O_2_ nano powder prepared using new precipitation method. Sens. Actuat. B Chem.

[87] Manorama SV, Izu N, Shin W, Matsubara I, Murayama N (2003). On the platinum sensitization of nanosized cerium dioxide sensors. Sens. Actuat. B Chem.

[88] Izu N, Shin W, Matsubara I, Murayama N (2004). Development of resistive oxygen sensors based on cerium oxide thick film. J. Electroceram.

[89] Alkemade UG, Schumann B (2006). Engines and exhaust after treatment systems for future automotive applications. Solid State Ionics.

[90] Moos R (2010). Catalysts as sensors—A promising novel approach in automotive exhaust gas aftertreatment. Sensors.

[91] Reiß S, Wedemann M, Moos R, Rösch M (2009). Electrical *in situ* characterization of three-way catalyst coatings. Top. Catal.

[92] Reiß S, Spörl M, Hagen G, Fischerauer G, Moos R (2009). Combination of wirebound and microwave measurements for *in situ* characterization of automotive three-way catalysts. IEEE Sens. J.

[93] Li H, Zhu Q, Li Y, Gong M, Chen Y, Wang J, Chen Y (2010). Effects of ceria/zirconia ratio on properties of mixed CeO_2_-ZrO_2_-Al_2_O_3_ compound. J. Rare Earths.

[94] Han Z, Wang J, Yan H, Shen M, Wang J, Wang W, Yang M (2010). Performance of dynamic oxygen storage capacity, water-gas shift and steam reforming reactions over Pd-only three-way catalysts. Catal. Today.

[95] Fischerauer G, Spörl M, Gollwitzer A, Wedemann M, Moos R (2008). Catalyst state observation via the perturbation of a microwave cavity resonator. Frequenz.

[96] Moos R, Wedemann M, Spörl M, Reiß S, Fischerauer G (2009). Direct catalyst monitoring by electrical means: An overview on promising novel principles. Top. Catal.

[97] Esper MJ, Logothetis EM, Chu JC (1979). Titania exhaust gas sensor for automotive applications. SAE Tech Paper.

[98] Fleischer M, Hanrieder W, Meixner H (1995). Oxygen sensor with semiconducting gallium oxide. European Patent EP 0464243.

[99] Sugie J (1994). Oxide semiconductor gas sensor. Japanese Patent H06-222,026.

[100] Frank J, Fleischer M, Meixner H (1996). Gas sensor. World Patent WO 9,608,712.

[101] Izu N, Shin W, Matsubara I, Murayama N (2004). Small temperature-dependent resistive oxygen gas sensors using Ce_0.9_Y_0.1_O_2−δ_ as a new temperature compensating material. Sens. Actuat. B Chem.

[102] Izu N, Shin W, Matsubara I, Murayama N, Oh-hori N, Itou M (2005). Temperature independent resistive oxygen sensors using solid electrolyte zirconia as a new temperature compensating material. Sens. Actuat. B Chem.

[103] Izu N, Nishizaki S, Itoh T, Shin W, Matsubara I, Murayama N (2007). Output evaluation of resistive oxygen sensor having Ce_0.9_Zr_0.1_O_2_ sensing material and Zr_0.8_Y_0.2_O_2-δ_ temperature compensating material in model exhaust gas. J. Ceram. Soc. Jpn.

[104] Izu N, Nishizaki S, Shin W, Itoh T, Nishibori M, Matsubara I (2009). Resistive oxygen sensor using ceria-zirconia sensor material and ceria-yttria temperature compensating material for lean-burn engine. Sensors.

[105] Merkle R, Maier J (2008). How is oxygen incorporated into oxides? A comprehensive kinetic study of a simple solid-state reaction with SrTiO_3_ as a model material. Angew. Chem. Int. Ed.

[106] Tragut C (1992). The influence of the surface transfer reaction on the response characteristics of resistive oxygen sensors. Sens. Actuat. B Chem.

[107] Dubbe A, Wiemhöfer HD, Göpel W (1994). Frequency response study of the kinetic behaviour of nernstian solid-electrolyte gas sensors by pressure modulation. J. Electroanal. Chem.

[108] Shin W, Izu N, Matsubara I, Murayama N (2004). Millisecond-order response measurement for fast oxygen gas sensors. Sens. Actuat. B Chem.

[109] Izu N, Shin W, Matsubara I, Murayama N (2004). Kinetic behavior of resistive oxygen sensor using cerium oxide. Sens. Actuat. B Chem.

[110] Izu N, Itoh T, Shin W, Matsubara I, Murayama N (2008). Evaluation of response characteristics of resistive oxygen sensors using Ce_0.9_Zr_0.1_O_2_ thick film by pressure modulation method. Sens. Actuat. B Chem.

[111] Wagner SF, Menesklou W, Schneider T, Ivers-Tiffée E (2004). Kinetics of oxygen incorporation into SrTiO_3_ investigated by frequency-domain analysis. J. Electroceram.

[112] Sahner K, Moos R, Izu N, Shin W, Murayama N (2006). Response kinetics of temperature independent resistive oxygen sensor formulations: A comparative study. Sens. Actuat. B Chem.

[113] Argirusis C, Jomard F, Wagner SF, Menesklou W, Ivers-Tiffée E (2011). Study of the oxygen incorporation and diffusion in Sr(Ti_0.65_Fe_0.35_)O_3_ ceramics. Solid State Ionics.

[114] Zheng H, Sørensen OT (2000). Integrated oxygen sensors based on Mg-doped SrTiO_3_ fabricated by screen-printing. Sens. Actuat. B Chem.

[115] Jonker GH (1968). Application of combined conductivity and seebeck-effect plots for analysis of semiconductor properties. Philips Res. Rep.

[116] Rettig F, Sahner K, Moos R (2005). Thermopower of LaFe_1−x_Cu_x_O_3−δ_. Solid State Ionics.

[117] Rettig F, Moos R (2010). α-iron oxide: An intrinsically semiconducting oxide material for direct thermoelectric oxygen sensors. Sens. Actuat. B Chem.

[118] Rettig F, Moos R (2007). Direct thermoelectric gas sensors: Design aspects and first gas sensors. Sens. Actuat. B Chem.

[119] Choi GM, Tuller HL (1988). Defect structure and electrical properties of single-crystal Ba_0.03_Sr_0.97_TiO_3_. J. Am. Ceram. Soc.

[120] Rettig F, Moos R (2008). Morphology dependence of thermopower and resistance in semiconducting oxides with space charge regions. Solid State Ionics.

[121] Rettig F, Moos R (2007). Direct thermoelectric hydrocarbon gas sensors based on SnO_2_. IEEE Sens. J.

[122] Rettig F, Moos R Thermoelectric gas sensors: Proof of reproducibility and geometry independency.

[123] Röder-Roith U, Rettig F, Röder T, Janek J, Moos R, Sahner K (2009). Thick-film solid electrolyte oxygen sensors using the direct ionic thermoelectric effect. Sens. Actuat. B Chem.

[124] Rettig F, Moos R (2009). Temperature modulated direct thermoelectric gas sensors: Thermal modeling and results for fast hydrocarbon sensors. Meas. Sci. Technol.

